# The Effect of Oral Laxatives on Rectal Distension and Image Quality in Magnetic Resonance Imaging of the Prostate

**DOI:** 10.7759/cureus.35539

**Published:** 2023-02-27

**Authors:** Robert W Foley, Hend Komber, Pia Charters, Noor Ali, Nick Burns-Cox, Paul R Burn

**Affiliations:** 1 Department of Diagnostic Imaging, Taunton and Somerset NHS (National Health Service) Foundation Trust, Taunton, GBR

**Keywords:** artefact, laxatives, bowel preparation, magnetic resonance imaging, prostate neoplasms

## Abstract

Introduction

Increasing rectal size is associated with increased artefacts on magnetic resonance imaging (MRI) of the prostate and has the potential to degrade image quality. The objective of this study was to analyse the effect of oral laxative medication on rectal distension and image quality in prostate MRI.

Methods

Eighty patients prospectively received either 15 mg of oral senna (laxative group) or no medication (control group). Patients underwent prostate MRI according to standard local protocol and seven rectal dimensions on axial and sagittal images were measured. A subjective assessment of rectal distension was also made using a five-point Likert scale. Finally, artefacts on diffusion-weighted sequences were assessed using a four-point Likert scale.

Results

There was a small reduction in rectal diameter on sagittal images in the laxative group compared to the control group, with mean diameters of 27.1 mm and 30.0 mm respectively, p=0.02. There was no significant difference in rectal measurements of anteroposterior diameter, transverse diameter, or rectal circumference on axial imaging. Subjective scoring also demonstrated no significant difference in diffusion-weighted imaging quality between the laxative group and control group, p=0.82.

Conclusion

Bowel preparation with the oral laxative, senna, provided only a marginal decrease in rectal distension on one measure and no reduction in artefacts on diffusion-weighted sequences. The findings of this study do not support the routine use of this medication in patients undergoing prostate MRI.

## Introduction

Prostate cancer incidence and burden of disease are increasing globally [1]. In the United Kingdom (UK), prostate cancer is the most common cancer in men and a leading cause of cancer-related mortality [2]. Magnetic resonance imaging (MRI) has become the gold standard imaging modality for patients under investigation for prostate cancer prior to biopsy [3,4].

The accurate implementation of pre-biopsy prostate MRI is dependent upon the quality of the images that are produced. One common cause of degraded image quality on prostate MRI is artefacts from gas within the rectum, which can cause significant degradation of the diffusion-weighted imaging (DWI) sequences in particular [5,6]. We have previously demonstrated that patients with reduced rectal distension are significantly more likely to have images of diagnostic quality [7], which may lead to more accurate identification of prostate cancer [6]. Techniques to reduce rectal distension may therefore improve the overall quality of a pre-biopsy MRI service.

One method to attempt to reduce rectal distention is administration of the oral laxative medication, senna. Senna is a stimulant laxative used for the treatment of constipation, which acts to promote peristalsis and evacuation. This laxative has the potential to reduce the size of the rectum, reduce artefacts, and lead to superior image quality in prostate MRI. The objective of this study was to analyse the effect of the oral laxative, senna, on rectal distension and image quality on prostate MRI.

## Materials and methods

Study cohort

The study population consisted of patients under clinical investigation for suspected prostate cancer in a urology outpatient setting in Taunton and Somerset NHS (National Health Service) Foundation Trust, Taunton, England. This study received ethical approval from the Taunton and Somerset NHS Foundation Trust Institutional Review Board and all patients provided written informed consent. Consecutive patients referred for prostate MRI were given alternately either oral laxative (laxative arm) or no medication (control arm). Oral laxatives were provided in the form of two 7.5mg tablets of SenEase (active ingredient: sennoside; Sigma PLC, Hertfordshire, UK). Patients were given an information leaflet and, for those in the laxative arm, instructed to take the medication at either 8pm the previous day for a morning scan, or 8am on the same day for an evening scan. All patients were also advised to empty their bowels prior to the study. During their MRI attendance, patients were asked to record the time that they last opened their bowels. Those in the laxative arm were asked to record the time at which they took their medication. Patients were excluded from the study if any of the following were present: a known allergy to senna, known or suspected intestinal obstruction, or undiagnosed abdominal pain. Two patients were excluded from the DWI analysis as each patient had bilateral hip prostheses leading to images of non-diagnostic quality.

MRI acquisition

Patients underwent MRI at 1.5T (MAGNETOM Aera, Siemens Healthineers, Erlangen, Germany) using a phased array coil over the pelvis. The scanning protocol (compliant with Prostate Imaging - Reporting and Data System (PI-RADS®) v2.1, American College of Radiology, Reston, Virginia, United States) included sagittal, axial and coronal T2-weighted (T2W) sequences (field of view 200-240mm, slice thickness 3.5-4mm) of the prostate [8]. Axial DWI multi-b sequence (b50 and b800) and separate b1400 sequence (both fields of view 250mm, slice thickness 4mm) (acquired as free-breathing echo planar imaging sequence with spectral fat suppression) and fat-suppressed, post-gadolinium, dynamic contrast-enhanced images were also acquired. Patients were given 20 mg intramuscular Buscopan (hyoscine butylbromide) to reduce bowel peristalsis immediately prior to the scan (if no contraindications).

MRI interpretation

**Figure 1:**
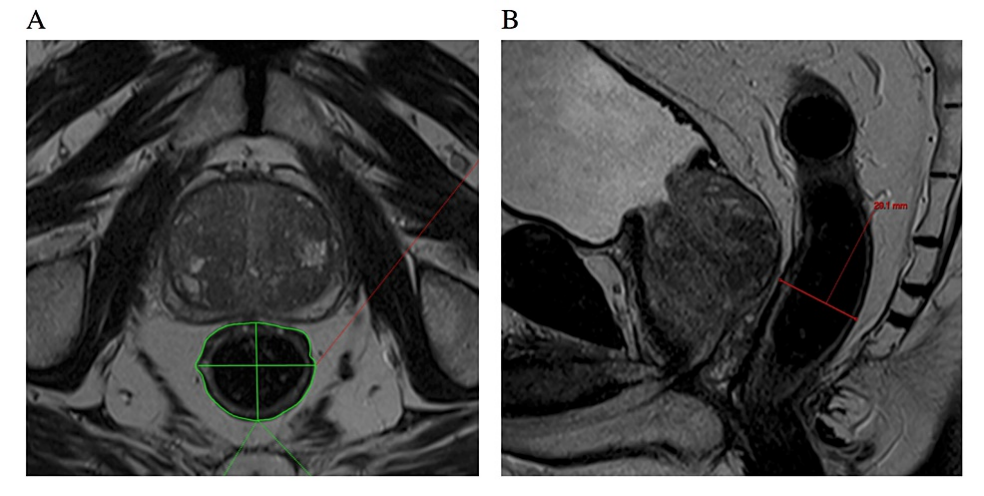
Axial T2 image (A) demonstrating the measurement of the anteroposterior diameter, transverse diameter, and area of the rectum on the slice with maximal prostatic dimensions. Sagittal T2 image (B) demonstrates the measurement of maximal sagittal rectal diameter perpendicular to the rectal wall.

Diameter and area measurements of the rectum were carried out by two radiology registrars with three years and four years of radiology experience, respectively, each measuring an equal number of patients in both the laxative arm and the control arm. These measurements were then reviewed by a consultant radiologist with 15 years of experience. Both observers were blinded as to which arm the patient was in, as well as to the clinical radiology report. Assessment and grading of DWI sequences were undertaken by a consultant radiologist with 15 years of experience. For each scan, seven objective measurements were made. Firstly the axial T2W image subjectively demonstrating the greatest area of the prostate was selected and on this, the anteroposterior (AP), transverse diameter, and area of the rectum were measured (the latter using a freehand region-of-interest tool to draw around the rectal circumference) (Figure 1A). These three measurements were then repeated on the axial T2W image subjectively demonstrating the greatest area of the rectum, while also still including prostate tissue. Finally, on the T2W sagittal sequence, the image subjectively showing the greatest rectal area was selected and the maximum AP diameter, perpendicular to the rectal posterior wall, was measured (Figure 1B).

**Figure 2:**
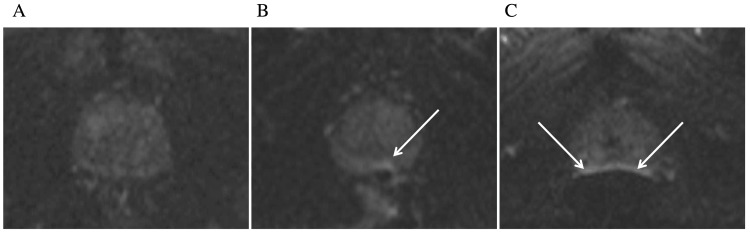
Axial DWI images demonstrate none (A), mild (B), and moderate (C) artefact secondary to rectal gas. DWI: diffusion-weighted imaging

An overall subjective assessment of rectal distension was also performed using a five-point Likert scale, as per Padhani et al. [9]; 1=no stool/gas, 2=minimal, 3=small amount, 4=moderate, 5=large amount of stool/gas. Finally, an assessment of the degree of artefact on DWI sequences was undertaken using a four-point Likert scale, as per Caglic and colleagues [6]; 1=none, 2=mild, not/mildly impacting diagnosis, 3=artefact moderately impacting diagnosis, 4=marked artefact/non-diagnostic (Figure 2).

Statistical analysis

Comparison of variables was undertaken between the intervention and control arms of the study. Variables were analysed for normality using the Shapiro-Wilk test. The means of variables that met the normality assumption were compared with the Student’s t-test, while those that did not were analysed with the Wilcoxon-Rank Sum test. Subjective scores for rectal distension using the Likert scale were dichotomised into mild rectal loading (scores 1, 2, and 3) or significant rectal loading (scores 4 and 5), and differences between the laxatives and control group were analysed using the Fisher’s exact test. DWI quality was also compared using the Wilcoxon-Rank Sum test. A p-value of <0.05 was considered statistically significant. Statistical analysis was carried out in R version 3.3.3 [10].

## Results

The cohort consisted of 80 men, 40 of whom received the oral laxative and 40 who did not. The clinical characteristics of the study cohort are demonstrated in Table 1. The mean age in the laxative and control arms were similar, as were the rates of Buscopan usage and the proportion of patients who opened their bowels on the day of the MRI scan. Those patients in the laxative group were likely to open their bowels closer to their MRI scan, 4.0 hours prior compared to 4.8 hours prior in the control group; however, this difference was not statistically significant (p=0.09).

**Table 1:** Characteristics of the Study Cohort Values are displayed as mean +/- standard deviation unless otherwise specified

	All	Laxative Group	Control Group
Age	68.2 +/- 7.5	68.2 +/- 7.4	68.0 +/- 7.6
Buscopan, n (%)	74 (93)	37 (93)	37 (93)
Bowels open the day of MRI, n (%)	76 (95)	38 (95)	38 (95)
Bowels opening to MRI scan (hours)	4.4 +/- 4.9	4.0 +/- 3.9	4.8 +/-5.7
Hours from senna to bowels opening		8.6 +/- 4.4	
Hours from senna to MRI scan		11.6 +/- 3.7	

**Figure 3:**
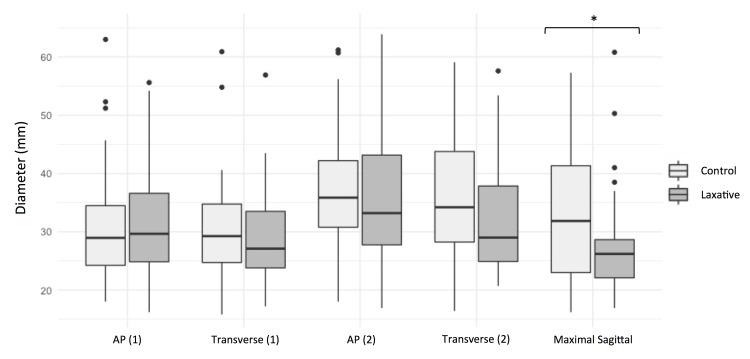
The rectal measurements in the laxative group (dark grey) and control group (light grey) are illustrated. The anteroposterior and transverse diameters are shown for the two axial slices analysed. The significant reduction in maximal diameter measured on the selected sagittal slice is also illustrated.

The mean rectal measurements for the total cohort, laxative group, and control group are given in Table 2. On the two axial images analyzed, there was no significant difference in AP diameter, transverse diameter, or rectal circumference. However, on sagittal images, there was a small reduction in the maximal rectal diameter in the laxative group compared to the control group, with mean diameters of 27 mm and 30 mm, respectively, which was statistically significant (p = 0.02). The average rectal diameters in the laxative and control groups are illustrated in Figure 3.

**Table 2:** Objective Measurements of Rectal Distension All values are displayed as mean +/- standard deviation * Wilcoxon Rank Sum test † Student paired T-test

	All	Laxative Group	Control Group	p value
Axial Slice 1				
Anteroposterior diameter (mm)	31.2 +/- 9.9	31.5 +/- 9.7	30.9 +/- 10.2	0.67*
Transverse diameter (mm)	30.4 +/- 10.2	30.5 +/- 11.5	30.4 +/- 8.7	0.60*
Area (mm^2^)	779 +/- 495	798 +/- 553	769 +/- 437	0.95*
Axial slice 2				
Antero-posterior diameter (mm)	36.2 +/- 10.0	35.5 +/- 10.3	36.8 +/- 9.8	0.56^†^
Transverse diameter (mm)	35.1 +/- 12.1	34.0 +/- 12.6	36.2 +/- 11.5	0.26*
Area (mm^2^)	1023 +/- 595	980 +/- 632	1066 +/- 561	0.44*
Sagittal slice				
Maximal diameter (mm)	30.0 +/-10.3	27.1 +/- 8.8	32.9 +/- 10.8	0.02*

The results of the subjective assessment of rectal distension are demonstrated in Table 3. Following dichotomisation of the variables there was no significant difference in subjective scoring, with the laxative group having 22 patients (55%) with small volume and 18 patients (45%) with large volume distension, compared to 24 (60%) small volume and 16 (40%) large volume distension patients in the control group, p = 0.82.

**Table 3:** Subjective Assessment of Rectal Distension

Grading Scale (Grade 1-3 = small volume; Grade 4-5 = large volume)	n
1 (no stool/gas)	7 (18%)
2 (minimal)	13 (33%)
3 (small amount)	2 (5%)
4 (moderate)	8 (20%)
5 (large)	10 (25%)

There was no statistically significant difference in DWI image artefact between the laxative group and the control group, p = 0.89. In the laxative group, there were 25 patients (64%) with no DWI artefact, 12 patients (31%) with mild artefact, and one patient with moderate artefact (3%). In the control group, there were 26 patients (67%) with no DWI artefact, 12 patients (31%) with mild artefact, and two patients with moderate artefact (6%).

## Discussion

The results of the present study do not demonstrate conclusive evidence for a reduction in rectal distension following senna laxative administration. One measurement (the sagittal rectal diameter) demonstrated a small statistically significant reduction in the laxative group compared to the control group; however, there was no significant difference for the other six measurement criteria. The subjective overall assessment of rectal distension also demonstrated no difference between those taking senna and the control group. There was also no reduction in DWI image artefacts in the senna group.

Although the findings of the present study do not support the routine use of oral senna, it is possible that a more intensive regimen of oral laxatives or an alternative method of bowel preparation would be more effective in decreasing rectal size prior to MRI. The decision as to what method to use is a difficult one.

In a systematic review of patients prior to prostate radiotherapy by McNair et al. and a further review by Bayles and colleagues, a wide variety of strategies have been discussed with no evidence to support one regimen over another [11,12]. Strategies that have demonstrated some success include dietary changes, enemas, and rectal emptying techniques. Nichol et al. analysed the effect of a dietary and oral laxative regimen upon rectal distension in patients undergoing MRI prior to prostate radiotherapy [13]. This study demonstrated that the use of this regimen did not lead to a significant reduction in the rectal area.

The role of an enema prior to MRI was investigated in a double-blinded prospective trial of 60 patients by Lim et al. [14]. Thirty-two patients underwent MRI without bowel preparation, while 28 patients utilised a self-administered enema. The enema group was demonstrated to have significantly less rectal stool in comparison to the controls. However, rectal distension was only analysed subjectively, using a five-point Likert scale. The lack of an objective measurement of rectal size means that care must be taken when interpreting these results. A subsequent paper from Coskun and colleagues also demonstrated that in prostate MRI, rectal diameter was significantly decreased in patients receiving a micro-enema compared to controls, with measurements of 24.8 mm and 26.7 mm, respectively [15]. The enema was administered 12 hours prior to the MRI.

Rectal distension may degrade MRI image quality either via increased rectal motion or due to increased susceptibility artefacts on DWI sequences because of the presence of increased rectal gas. Padhani et al., using cine MRI, showed a relationship between rectal distension and rectal movement [9]. Caglic et al., in a retrospective study of 173 patients, found increased rectal size was strongly correlated with decreased DWI image quality, and the authors concluded by recommending the consideration of bowel preparation of patients prior to prostate MRI [6]. However, the results of the present study did not demonstrate any improvement in DWI image quality following the administration of senna.

van Griethuysen et al. assessed the effect of micro-enema administration given 15 minutes prior to MRI of the rectum on reducing gas artefact of diffusion sequences [16]. Of 355 studies, 24.3% of scans had significant artefacts in patients without bowel preparation, compared to just 3.7% in patients receiving the micro-enema. In contradistinction, the studies of Lim et l. [14] and Coskun et al. [15] did not find significant improvements in image quality following micro-enemas, although in these cases the enema was administered the morning before and 12 hours before the MRI, respectively.

In the present study, a possible explanation for the lack of efficacy of oral laxatives is the advice we gave to all study participants to empty their bowels immediately prior to scan acquisition, as per PI-RADS® v2 guidance [8]. We have demonstrated that the average time between bowel opening and the MRI was not statistically different between the intervention and control groups. It is possible, therefore, that this simple advice prior to the scan is more important than the use of bowel preparation.

Limitations

There are several limitations to the present study. Firstly, there is a relatively small number of patients; however, it still compares favourably with many of the previous publications on this topic. Secondly, this was not a double-blind study. In particular, patients who received senna may have, by choice, modified their bowel habits irrespective of the effect of the medication, compared to the control group. Thirdly, although we made several different rectal size measurements, including both diameter and circumference, a more in-depth analysis of rectal volume using volumetric software was not performed.

## Conclusions

We found that only one of the seven rectal measurements analysed showed a small significant difference in patients taking oral laxatives compared to controls. While there was no difference in subjective rectal size or DWI image quality. Therefore, this study does not conclusively demonstrate a reduction in rectal size following senna administration in patients undergoing prostate MRI, nor does the study demonstrate an improvement in image quality with this regimen. The routine use of this bowel preparation to reduce potential artefact from the rectum is therefore not supported.
